# 665. Postoperative Complications of Soft Versus Rigid Based Tissue Expanders in Burn and Soft Tissue Reconstruction

**DOI:** 10.1093/jbcr/irag033.531

**Published:** 2026-04-06

**Authors:** Inbal Alkalay, Alex Cohen, Tomer Kerman, Dor Halpern, Noa Sabag, Gefen Bloom, Ofir Ron, Roy Grinberg, Eliran Jacobi, Subhi Hakrush, Meir Retchkiman, Eldad Silberstein, Yaron Shoham

**Affiliations:** Soroka University Medical Center, Be’er Sheva, HaDarom; Soroka University Medical Center, Be’er Sheva, HaDarom; Soroka University Medical Center, Be’er Sheva, HaDarom; Soroka University Medical Center, Be’er Sheva, HaDarom; Soroka University Medical Center, Be’er Sheva, HaDarom; Soroka University Medical Center, Be’er Sheva, HaDarom; Soroka University Medical Center, Be’er Sheva, HaDarom; Soroka University Medical Center, Be’er Sheva, HaDarom; Soroka University Medical Center, Be’er Sheva, HaDarom; Soroka University Medical Center, Be’er Sheva, HaDarom; Soroka University Medical Center, Be’er Sheva, HaDarom; Soroka University Medical Center, Be’er Sheva, HaDarom; Soroka University Medical Center, Be’er Sheva, HaDarom

## Abstract

**Introduction:**

Tissue expanders are a well-established method in reconstructive surgery. Tissue expander reconstructions may be divided between breast-related and non-breast related reconstructions. While prior studies have investigated diverse aspects of expanders' complications in both indications, there is a paucity of published research regarding the impact of expander base rigidity on complications. This study aims to investigate the association between expander base type rigidity and complications in non-breast reconstructions.

**Methods:**

We conducted a retrospective analysis of non-breast-related tissue expansion procedures in patients aged 6 months to 76 years, treated at a tertiary medical center from 2000 to 2021. We compared soft-based and rigid-based expanders, analyzing demographic factors, anatomical placement, shape, and indications using mixed models and multivariate Poisson regression to estimate the risk ratio (RR) and adjusted for confounders.

**Results:**

A total of 86 patients underwent non-breast-related tissue expansion procedures, utilizing 186 expanders, of which 130 were rigid-based and 56 soft-based. Post-burn reconstruction was the indication in 54% of rigid-based and 53% of soft-based expanders used. The groups did not differ in demographic characteristics. Univariate analysis revealed complication rates of 18% for the soft-based and 50% for the rigid-based groups (*p*<.001). Another significant difference was found specifically in major complications (16% vs. 45% respectively, *p*<.001), with wound infection and expander exposure as the most significant complications (5.4% vs. 25.0%, *p*=.045 and 5.4% vs. 23.0%, *p*=.013, respectively). We did not find associations between the geometric shape or anatomical placement of the tissue expanders and complication rates.

**Conclusions:**

Soft based tissue expanders had a significantly lower rate of complications as compared to rigid based tissue expanders, particularly in major complications including wound infection and expander exposure.

**Applicability of Research to Practice:**

Based on the results of this retrospective study we recommend on preferring soft based tissue expanders for burns and other non-breast tissue expander reconstructions.

**Funding for the study:**

N/A.

